# Embodied large language models enable robots to complete complex tasks in unpredictable environments

**DOI:** 10.1038/s42256-025-01005-x

**Published:** 2025-03-19

**Authors:** Ruaridh Mon-Williams, Gen Li, Ran Long, Wenqian Du, Christopher G. Lucas

**Affiliations:** 1 University of Edinburgh, Edinburgh, UK; 2 Massachusetts Institute of Technology, Boston, MA USA; 3 Princeton University, Princeton, NJ USA; 4 Alan Turing Institute, London, UK

**Keywords:** Engineering, Computer science

## Abstract

Completing complex tasks in unpredictable settings challenges robotic systems, requiring a step change in machine intelligence. Sensorimotor abilities are considered integral to human intelligence. Thus, biologically inspired machine intelligence might usefully combine artificial intelligence with robotic sensorimotor capabilities. Here we report an embodied large-language-model-enabled robot (ELLMER) framework, utilizing GPT-4 and a retrieval-augmented generation infrastructure, to enable robots to complete long-horizon tasks in unpredictable settings. The method extracts contextually relevant examples from a knowledge base, producing action plans that incorporate force and visual feedback and enabling adaptation to changing conditions. We tested ELLMER on a robot tasked with coffee making and plate decoration; these tasks consist of a sequence of sub-tasks from drawer opening to pouring, each benefiting from distinct feedback types and methods. We show that the ELLMER framework allows the robot to complete the tasks. This demonstration marks progress towards scalable, efficient and ‘intelligent robots’ able to complete complex tasks in uncertain environments.

## Abstract (web summary)

To function in the real world, autonomous robots will have to respond to unanticipated situations. A vision-language-model-based approach is proposed to solve long-horizon robotic tasks, which can adapt to a dynamic environment.

## Main

If Deep Blue (the first computer to win a chess match against a reigning world champion) was truly intelligent, then should it not be able to move its own pieces when playing chess? Intelligence is a multifaceted construct and, thus, difficult to define. Consequently, human intelligence and its assessment is a controversial topic^1^. However, there is a growing consensus that human intelligence is best understood as ‘embodied cognition’, where attention, language, learning, memory and perception are not abstract cognitive processes constrained to the brain but intrinsically linked with how the body interacts with its surrounding environment^2,3^. Indeed, there is growing evidence that human intelligence has its ontological and phylogenetic foundations in sensorimotor processes^4^.

Embodied cognition has theoretical implications for ‘machine intelligence’ as it suggests that machines will be unable to demonstrate some aspects of intelligence if ‘cognitive’ processes are not embedded in a robotic device. This is a conjecture that is still to be tested, but ‘intelligent robots’ provide an effective way of exploring various hypotheses concerning human intelligence and advancing the field of machine intelligence. More practically, effective human–robot collaboration will ultimately require robots to at least approximate ‘human-like’ capabilities. Thus, a reasonable expectation of future ‘intelligent machines’ is that they will have the potential to perform abstract cognitive computations as they skilfully interact with objects and humans within their environment^5^.

So far, parallel streams of activity have advanced: (1) the sensorimotor abilities of robots and (2) artificial intelligence^6^. We set out to test the hypothesis that these approaches can now be combined to create a step change in the ability of robots to show human-like intelligence. We further hypothesized that integrating (1) and (2) would allow robots to undertake the type of complex tasks that are practically useful in a wide range of settings but currently outwith the capability of robotic systems. Consider a scenario in which someone returns home feeling fatigued and thirsty. A robot with a sophisticated manipulation system is situated in the homeowner’s kitchen and is instructed to prepare a drink. The robot decides that a reinvigorating cup of coffee needs to be made and handed to their carbon companion. This task—straightforward for humans—encompasses a series of challenges that test the limits of current robotic capabilities^7–11^. First, the robot must interpret the information it receives and analyse its surroundings. Next, it may need to search the environment to locate a mug. This could involve opening drawers with unspecified opening mechanisms. Then, the robot must measure and mix the precise ratio of water to coffee. This requires fine-grained force control and adaptation to uncertainty if, for example, the human moves the location of the mug unexpectedly^9,12^. This scenario is a canonical example of the multifaceted nature of complex tasks in dynamic environments. Robotic systems have traditionally struggled with these tasks because they have been unable to follow high-level commands, have relied on preprogrammed responses and lack the flexibility to adapt seamlessly to perturbations^13,14^.

Reinforcement learning and imitation learning have demonstrated the effectiveness of interaction and demonstration in teaching robots to perform complex tasks. These approaches are promising^15^, but often struggle with adaptation to novel tasks and coping with diverse scenarios. Imitation learning also faces challenges when a robot needs to adapt to new contexts^16–23^. Nature-inspired machine intelligence provides a potential solution to these challenges. The sophistication of human manipulation is due, in part, to the type of cognitive processes that are captured artificially by large language models (LLMs)^24–26^. LLMs offer a way to process complex instructions and adapt actions accordingly because of their advanced contextual understanding and generalization abilities^27,28^.

A large body of recent research has used LLMs for short-horizon tasks^15,27,29^. For instance, VoxPoser utilizes LLMs to perform a variety of everyday manipulation tasks^15^. Similarly, Robotics Transformer (RT-2) leverages large-scale web and robotic learning data, enabling robots to perform tasks beyond the training scenario with remarkable adaptability^29^. Hierarchical diffusion policy introduces a model structure to generate context-aware motion trajectories, which enhances task-specific motions from high-level LLM decision inputs^30^. However, challenges remain in effectively integrating LLMs into robotic manipulation. These challenges include complex prompting requirements, a lack of real-time interacting feedback, a dearth of LLM-driven work exploiting the use of force feedback and inefficient pipelines that hinder the seamless execution of tasks^15,31^. Moreover, current approaches have neglected the application of retrieval-augmented generation (RAG)^32^ in robotics despite RAG’s potential to continually update and refine robot knowledge with relevant and accurate examples (and increase the knowledge base without impacting performance). Robot capacity is also limited because force and visual feedback are typically not integrated in robot sensorimotor control^15,33^. This integration is crucial in scenarios such as pouring water into a moving cup, where vision is necessary to track the cup and force feedback is needed for pouring the desired amount of water when vision is occluded^16,34,35^. Thus, there is a need for an innovative approach in robot manipulation that combines the latest artificial ‘cognition’ with integrated ‘sensorimotor’ visual and force feedback capabilities to effectively execute actions in the face of uncertainty. Supplementary Section 1 provides more background on state-of-the-art approaches and their current limitations^36–53^.

**Fig. 1:**
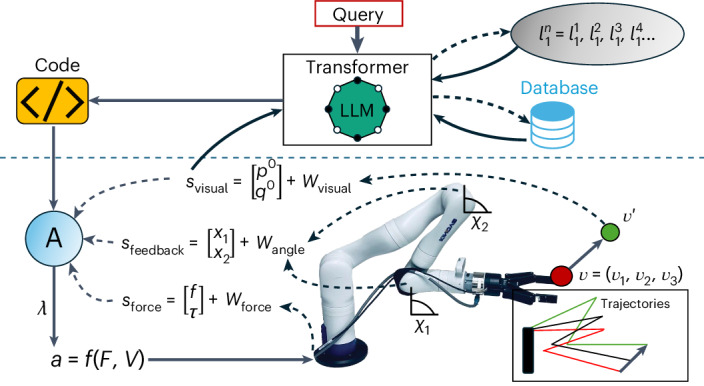
Schematic of the system framework. The schematic illustrates the system framework, showing the high-level (above the blue dashed horizontal line) and low-level (below the blue dashed horizontal line) system architecture. User queries are fed into a transformer via voice recognition software. The transformer (GPT-4) takes this input and integrates it with (i) an image (*C*) of the environment (via an azure Kinect depth camera); (ii) knowledge base of code examples, including various functions stored in a database. The transformer can decompose the higher-order abstracted task into actionable high-level sub-tasks, retrieve relevant code examples from the knowledge base, adapt them and write Python (v.3.8) code tailored to these tasks. The resultant code is then sent to the robot controller (A). The controller processes the code and sends control signals (*λ*) to the robot. The actions (*a*) are controlled with force (*F*) and vision (*V*) feedback. The model uses vision to identify the properties of different objects (for example, pose *X* of a coffee cup), so it can grasp objects accurately. The robot uses force (*f*) and torque (*τ*) feedback (available via an ATI force transducer) to manipulate objects skilfully (for example, determine how much water to pour). Feedback is necessary due to noise within the vision signal (*η*_vision_), the robot joint angles (*η*_angle_) and the force transducer signal (*η*_force_). The feedback updates the motion in the ROS to achieve the desired goal through velocity commands of both linear (*v*_*xyz*_) and angular (*v*_*rpy*_) velocities. These commands generate trajectories based on appropriate forces and spatiotemporal patterns to achieve the sub-goals. The use of feedback loops, including 40-Hz updates of the end-effector position (*p*) and orientation (*q*), allow the robot to respond to disturbance (for example, the robot tracking a cup to determine its new position after it is moved by the user).

Embodied LLM-enabled robot (ELLMER) is a framework that integrates approaches in artificial intelligence and sensorimotor control to create a step change in robotic capabilities. Its usefulness arises from its combined use of vision and force for sensorimotor feedback control uniquely coupled with the cognitive capabilities afforded through an integrated LLM combined with RAG and a curated knowledge base. We hypothesized that ELLMER would allow a robot to make a cup of coffee for a human. We tested this hypothesis using a seven-degrees-of-freedom Kinova robotic arm to execute the complex, force-intensive task in an uncertain environment, leveraging integrated force and vision feedback. The overall system diagram is presented in Fig. 1.

## Results

### Language integration for abstract reasoning

We found that our approach allowed the robot to respond to an abstract high-order verbal prompt (‘I’m tired, with friends due for cake soon. Can you make me a hot beverage, and decorate the plate with a random animal of your choice’) and complete the desired task of making a hot drink and drawing a shape on a plate. The video demonstrating this interaction is provided in Supplementary Video 1. The robot responded to the prompt with the following sound output: ‘I’m setting things up to make a hot beverage, and also to decorate the plate with a random animal drawing. First, I’ll find a mug then scoop coffee and pour hot water. After that I’ll take an item, likely a pen, to draw a random animal on the plate. It sounds like a fun and cosy time with cake soon.’. In particular, the robot chose to make a cup of coffee when it responded to the request for a hot beverage for a tired human, and was able to decompose the higher-order behaviour into a series of sub-tasks.

The integration of GPT-4 (ref. ^54^) was found to equip the robot with the desired capacity for abstract reasoning. GPT-4 is a language model that enables a robot to process user queries and environmental data to break down tasks into actionable steps. Our system was able to generate code and execute actions with force and vision feedback, effectively providing the robot with a form of intelligence. Our methodology was successful in creating a custom GPT-4 (refs. ^54,55^) with a comprehensive database of flexible motion examples. The database successfully incorporated pouring, scooping, drawing, handovers, pick and place, and opening doors.

We found that the robot could identify and extract relevant examples for the downstream task using RAG. We explored various approaches to determine how intelligent machines could make the best use of RAG via our framework. These approaches included customizable open-source methods, such as Haystack^56^ and Vebra^57^, as well as proprietary technologies such as Azure Cloud AI. We found that all of these approaches were viable. In our experiment, we chose the simplest method: logically organizing our curated knowledge base in a markdown file and uploading it to the custom GPT API via the ‘Knowledge’ feature in the GPT’s platform. This allowed the platform to automatically handle the retrieval processes and select between semantic search (returning relevant text chunks) or document review (providing complete documents or sections from larger texts). We chose this solution as it provided a state-of-the-art embedder and model, gave ease of use and was able to consistently produce good performance in our task. However, our framework allows the incorporation of a range of RAG techniques and ensures that the ‘intelligent robot’ is able to efficiently complete complex tasks. The curated knowledge base, combined with RAG, allowed the language model to access a large selection of low- and high-order functions, each with known uncertainties. Our tests showed that this capability enabled the robot to handle numerous scenarios effectively.

### Completing a complex task

**Fig. 2:**
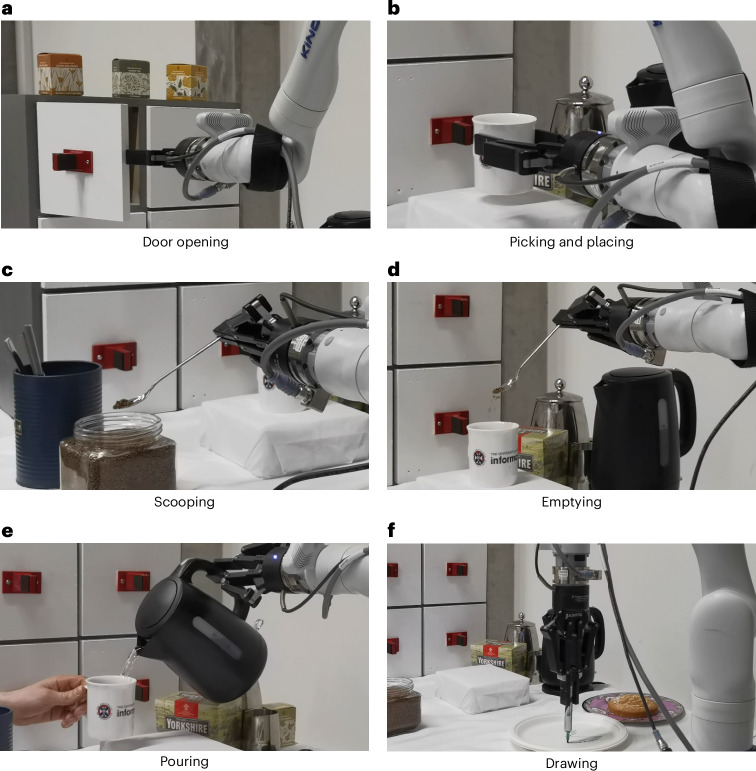
Kinova robot in action. **a**–**f**, Action shots of the Kinova Gen3 robot preparing coffee (**a**–**e**) and decorating a plate (**f**).

The robot was found to skilfully execute the high-level task specified by the user and was able to access a comprehensive motion primitive database. The database included a variety of flexible examples of specific motions and these were successfully carried out by the robotic arm (Fig. 2). Included in the database were examples of pouring liquids; scooping powders; opening doors with unknown mechanisms; picking up and placing objects; drawing any requested shape; conducting handovers; and moving in various directions, orientations or relative to specified objects. The robot was able to replicate and adapt the motions needed to execute the complex tasks requested by the user. The system enabled the robot to dynamically adjust to environmental variables and uncertainties. This enhanced the robot’s effectiveness in unpredictable conditions, and improved its flexibility and adaptability in the real-world setting.

### Zero-shot pose detection

We found that an Azure Kinect DK Depth Camera, set to a resolution of 640 × 576 px^2^ with a sample rate of 30 fps for depth sensing, was able to provide sufficient visual input for our method. We achieved calibration using a 14-cm AprilTag, and found that this allowed alignment between the camera and the robot’s base to an accuracy of less than 10^−6^. This setup enabled accurate object position detection within the scene. Grounded-Segment-Anything^58^ was successfully deployed for our language-to-vision module.

The vision system generated a three-dimensional (3D) Voxel representation that was effective at identifying object poses in our setup (the used Grounding DINO detection module achieved an average precision of 52.5 on the COCO zero-shot transfer benchmark). For example, we found that the module was able to correctly identify the white cup we used 100% of the time under our experimental conditions.

**Fig. 3:**
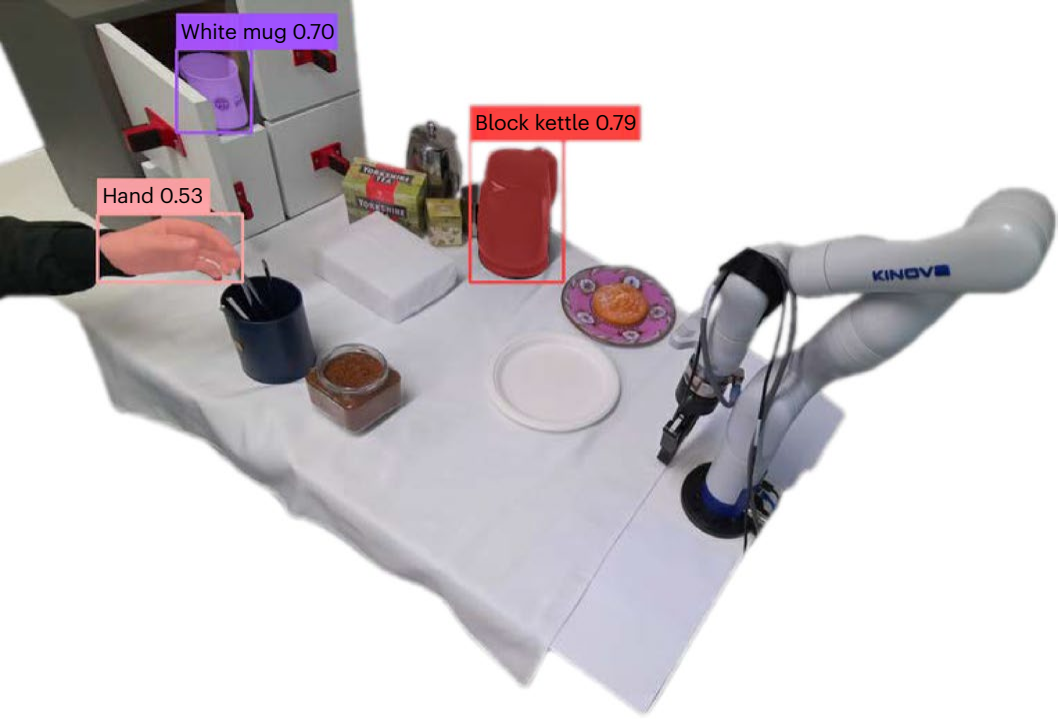
Vision detection module. Illustration of the zero-shot vision detection module identifying a hand, white mug and black kettle, as well as extracting target poses for robotic grasping.

The 3D Voxel representation contained the meshes of various objects. From these meshes, target poses were extracted at a frequency of 1/3 Hz. In principle, the system should have been able to detect any object. In the pilot work, however, we established that the system would not always accurately identify the different objects associated with making hot beverages. This was often due to confusion between objects with similar shapes or objects absent from the training dataset. We also found that occlusion caused by the robot’s end-effector could sometimes result in inaccuracies in object detection and lead to errors when we used highly cluttered environments. For example, the mean successful identification rate for a white cup was ~90% at occlusion ratios between 20% and 30%, but decreased substantially at higher occlusion ratios (for example, to ~20% for occlusion ratios between 80% and 90%). We anticipate that improvements in computer vision will enhance the ability of robots to deal with even the most visually complex environments. However, the performance of the vision system was impressive, and we found that our system could cope well with relatively unconstrained environments if the identified issues (for example, using out-of-distribution objects) were avoided (Fig. 3).

### Force feedback

We found that an ATI multiaxis force and torque sensor provided sufficient force feedback for skilful object interaction. The sensor provided six components of force and torque, and the forces exerted by the robot’s end-effector during task execution were successfully measured. We found that the sensor’s accuracy was within ~2% of the full scale at a sampling rate of 100 Hz.

**Fig. 4:**
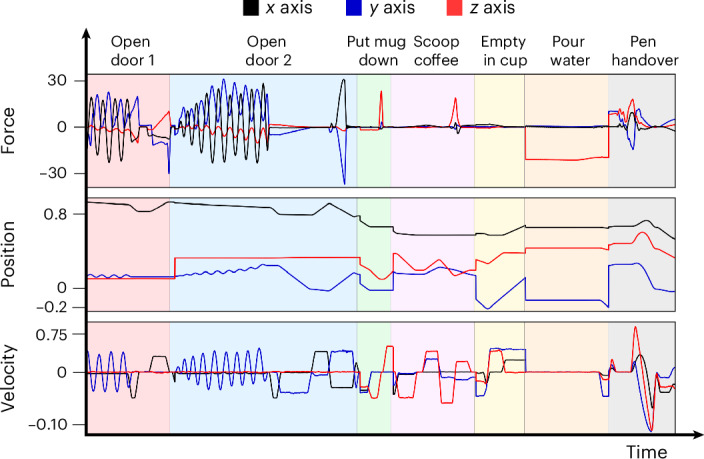
Force, velocity and position feedback. Force (N), velocity (m s^–1^) and position (m) plots during the robot’s coffee preparation, illustrating the diverse force feedback across the different motions. The drawing component has been omitted for clarity.

The robot was found to demonstrate a variety of motion dynamics accompanied by distinct types of force feedback during task execution. Figure 4 illustrates the forces experienced as the robot was preparing coffee and handing over a pen. As shown in Fig. 4, a diverse spectrum of external forces was handled across various tasks. For example, when putting down a mug, the peak upward force was used as an indicator of successful placement. By contrast, during drawer manipulation, the forces and torques along the *x* and *y* axes were critical, highlighting their importance for successful task execution. The variability in force feedback exemplifies the advantages of our scalable approach that adapts to the requirements of diverse motions.

The pouring accuracy achieved was ~5.4 g per 100 g at a pitch velocity of 4 m s^–1^. We assumed a quasi-static equilibrium to estimate the volume of water poured at any given moment. However, as the pitch velocity increased, the accuracy decreased, with errors approaching ~20 g s^–1^ at a pitch velocity of 30 m s^–1^. This decrease in accuracy can be attributed to the breakdown of the quasi-static assumption and the impact of the mass distribution of both pouring medium and container on measurement accuracy.

### Generating art

**Fig. 5:**
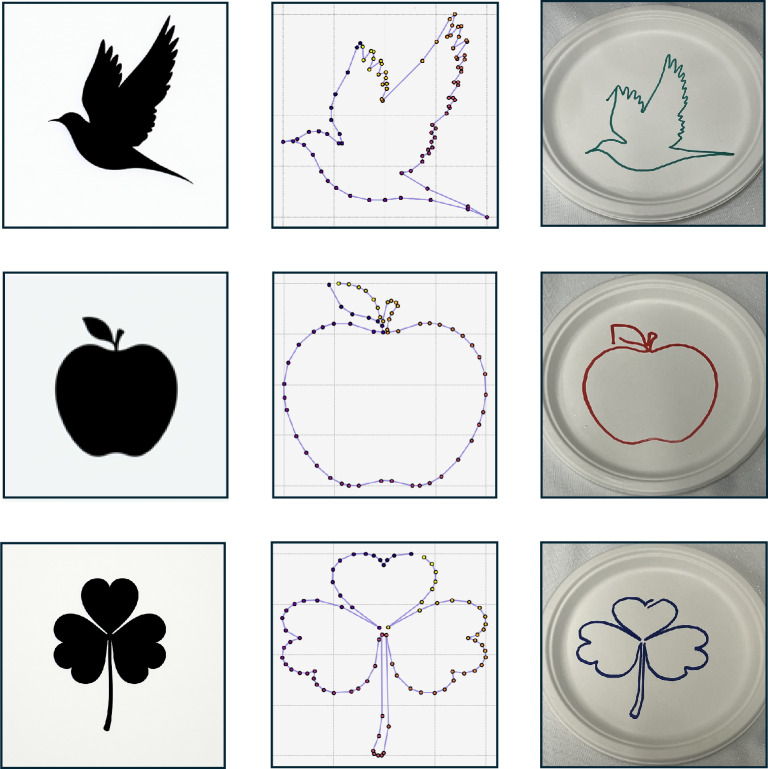
Drawing process visualization. Illustration of the drawing process across different queries. The top row shows the generated image, contour plot and drawing produced when instructed to create a ‘random animal’. The second row displays the corresponding outputs for a ‘random food’ and the third row illustrates the results for a ‘random plant’.

DALL-E^59^ was found to successfully produce an image from which we could derive a drawing trajectory. It was found that this enabled the robot to draw any design specified by the user. We found that DALL-E was able to create silhouettes based on keywords extracted from the user, such as ‘random bird’ or ‘random plant’. The silhouette’s outline was extracted and transformed to match the dimensions of the target surface. This allowed the robot to replicate the design on various physical objects (Fig. 5). We found that force feedback applied an even pen pressure when drawing, and this allowed control over the *z* component (Supplementary Section 2).

### Evaluation

We evaluated our method for generating robotic plans against VoxPoser, which does not utilize RAG or force feedback. To compare the methods, we prompted an LLM to generate 80 human-like queries, reflecting the range of tasks specified in the knowledge base. These queries were then used to generate robot plans. We compared the performance outcomes from using RAG (our method)—in which the knowledge base is dynamically integrated into the LLM’s decision-making process—to a baseline (VoxPoser) in which the knowledge base was statically incorporated into the LLM’s context window. It is important to note that the second approach lacks scalability and becomes impractical as the knowledge base expands.

We evaluated the results based on answer faithfulness, which assesses an answer’s truthfulness and accuracy (ensuring factual representation without fabrication or ‘hallucination’ errors). In our findings, using RAG improved the faithfulness of responses. For GPT-4 (gpt-4-0613), the faithfulness score increased from 0.74 to 0.88 with RAG. Similarly, GPT-3.5-turbo (gpt-3.5-turbo-0125) achieved 0.86 with RAG compared with 0.78 without it, and Zephyr-7B-beta saw an increase from 0.37 to 0.44. The improvement in faithfulness is particularly key for robotic applications, where accurate execution during physical interactions is essential.

## Discussion

We tested our methodology—the ELLMER framework—that combines techniques from artificial intelligence and robot manipulation to create an intelligent robot. Our approach successfully combined the cognitive abilities of LLMs with the sensorimotor skills of robots, enabling our robot to interpret a high-order verbal command and execute a complex long-horizon task while adeptly managing uncertainties. We used the LLM, augmented with feedback loops and RAG, to write expressive code and facilitate the manipulation sub-tasks required by the robot to achieve the high-level goal (making a hot beverage). ELLMER allowed real-time adaptation to environmental changes and leveraged a repository of precise solutions via RAG. This ensured accurate task execution and broad adaptability^32^.

ELLMER encoded known constraints into the code examples (‘motion functions’) and enabled rapid accommodation to numerous uncertainties, such as fluctuating ingredient quantities or opening unknown drawers—capabilities that other methods lack without extensive additional training^29,33,60,61^. The integration of vision, force and language modalities enhanced the manipulation performance. The force sensors improved task precision (for example, pouring a precise and accurate amount of liquid when vision was occluded), whereas the vision system identified object positions and movements. The language capabilities enabled the system to produce feedback within the code, which is critical for adjusting to new tasks. The curated knowledge base improved the LLM’s performance by tailoring information retrieval to the specific task specifications, and this ensured high-quality contextually relevant outputs. A curated knowledge base is a pragmatic element that enhances controllability, accuracy and scalability. In this context, RAG can be seen as providing a cultural milieu of knowledge from which a robot can draw. In particular, this mirrors the ‘intelligence’ afforded to humans through the cultural transmission of knowledge. Thus, our work shows that integrating advanced language models and sensorimotor control strategies allows robots to leverage the exponential advancements in LLMs, enabling more sophisticated interactions. This will usher in the next age of automation with unprecedented levels of autonomy and precision, accentuating the need to manage these advancements safely^62^.

ELLMER’s potential extends to creating intricate and artistic movements. For instance, a model like DALL-E allows trajectories to be derived from visual inputs and opens new avenues for robotic trajectory generation. This method can be widely applied in tasks such as cake decoration or latte art. In future work, incorporating queries and images will enable novel trajectory generation, allowing for increased versatility. Moreover, recent LLM enhancements are set to notably improve the fluidity and effectiveness of human–robot interactions. Our examples of coffee making and plate decoration represent only a subset of the complex task types that a sophisticated robot might be required to undertake. ELLMER is conducive to being scaled up, so it includes a wide range of possible long-horizon tasks. Thus, ELLMER could incorporate a database of feedback loops or ‘learning-from-demonstration’ examples to facilitate a wide variety of complex robotic manipulations.

ELLMER is based on two assumptions concerning computer vision: (1) the vision module accurately identifies and classifies objects within the scene and (2) a comprehensive affordance map of the utensils is available. We endowed our model with prior knowledge of the kettle, spoon and door handle affordances, but recent work suggests that affordances can be learned with minimal data^63,64^. Our focus was not on object detection, but we noted that detection response times hindered optimal performance. In addition, ELLMER could adjust to real-time changes but struggled with proactive adaptations (for example, task switching midway without prior programming). In future iterations, more frequent querying of the language model would allow the reassessment and modification of overall plans based on new inputs. We also note that there are still challenges that need to be addressed, such as the sophisticated modelling of complex force dynamics (for example, the forces on the end-effector as a function of the flow rate, container size and liquid viscosity) and the integration of spatial awareness tools (such as OctoMaps, a robotic library for a 3D occupancy map). Incorporating tactile sensors and using soft robotic techniques would improve the robot’s ability to apply appropriate forces without causing damage. ELLMER provides a flexible platform for incorporating these research developments, enabling robots to use ‘sensory’ feedback to interpret material properties and precisely tailor the forces they apply.

The current iteration of ELLMER allowed the robot to successfully complete a complex task in ‘one shot’. This provides a compelling picture of the capabilities of intelligent machines that combine sensorimotor capabilities with the abstract reasoning provided by LLMs. Nevertheless, we anticipate that the robot capacity will increase exponentially as the components combined within ELLMER become ever more refined. Our framework is hardware agnostic and can be easily customized with open-source RAG solutions like Haystack, supporting quick adjustments to embedders, retrievers, chunking techniques and LLMs. ELLMER offers a flexible framework for researchers to collaboratively develop intelligent machines. Supplementary Section 3 provides more information on ELLMER and future research.

The power of our approach lies in the embodiment of cognition through a framework that combines enhanced sensorimotor abilities with the cognitive reasoning capabilities of LLMs. Through this combination, ELLMER enables robots to explore and interact with their environment more effectively, emulating aspects of the connection between experience and action observed in human intelligence. This opens up opportunities for robots to gain a form of ‘physical intelligence’, where their exploration of the environment drives the sensorimotor learning process. In conclusion, ELLMER integrates language processing, RAG, force and vision to enable robots to adapt to complex tasks. It combines the following features: (1) interpreting high-level human commands, (2) completing long-horizon tasks and (3) utilizing integrated force and vision signals to manage noise and disturbances in changing environments. ELLMER allows methods such as reinforcement learning, imitation learning and flexible motion primitives to be combined holistically for enhanced adaptability and ‘robot intelligence’ in diverse and dynamic scenarios. It demonstrates that integrating the cognitive reasoning capabilities of LLMs with robots’ sensorimotor skills allows them to interpret and manipulate their environment and complete complex tasks through embodied machine intelligence.

## Methods

### Overview

The goal of the robot was to respond to high-level human commands in a dynamic environment, such as a home kitchen. We designed a realistic setting featuring items including a kettle, white mug, drawers, kitchen paraphernalia and a coffee pot. The scenario was designed to test the robot’s ability to perform diverse tasks in a realistic, although reasonably constrained, environment as it interacts with a human present. We assumed that robotic low-level control mechanisms managed obstacle avoidance. The pipeline consisted of a language-processing component for task execution, a vision system for pose detection and a force module for object manipulation. All of this was integrated within a robotic operating system (ROS) process.

**Fig. 6:**
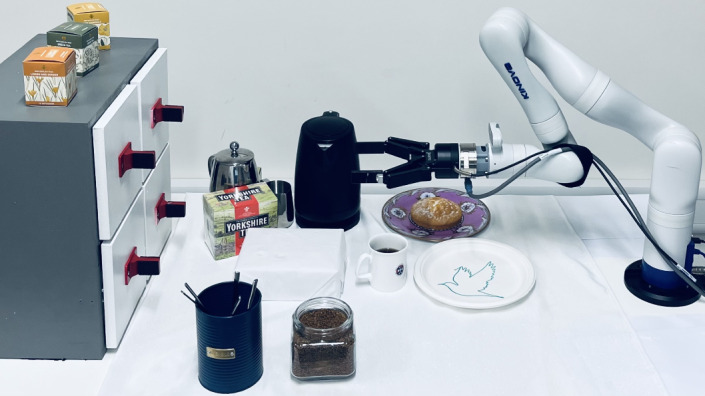
Coffee and plate decoration. Kinova Gen3 robot having prepared the coffee and decorated a plate.

Specifically, our approach built on the ‘code for dynamic policies’ approach^65^ that can facilitate adaptable robotic actions. In our implementation, we utilized GPT-4 and OpenAI’s RAG infrastructure. We leveraged LLMs’ capabilities using RAG^32^ to dynamically select and adapt the most suitable policy from a database or generate its own code based on relevant examples. In contrast to existing pure LLM-driven methods^25,27,29^, we integrated force and vision into the framework, allowing the system to adapt to a variety of complex tasks in dynamic settings. This approach equips the robotic system with the capacity for high-level contextual understanding^25^ and the proficiency to execute complex tasks with real-time feedback, ensuring accuracy and precision. The approach ensures that each action is aligned with the specific demands of the task and the environmental conditions (Fig. 6).

### Hardware and software

A Kinova seven-degrees-of-freedom robot was used. An Azure Kinect Sensor was used at a resolution of 640 × 576 px^2^ and 30 fps, along with an ATI multiaxis force sensor. A 140-mm Robotiq gripper was attached to the end of the robot. The force sensor was attached to the Robotiq gripper and Kinova arm using a 3D printed flange. A small cylinder was placed on the force sensor on the side closest to the gripper so that the movements of the gripper would not touch the force sensor, leading to readings being inaccurate. A Dell desktop computer with an Intel Core i9 processor with an NVIDIA RTX 2080 graphics-processing unit was used and connected to the robot with an Ethernet cable. Similarly, both Azure cameras were attached to the desktop. Ubuntu 20.04 and the ROS were used. Our code relied on the Kinova ROS Kortex library. The NVIDIA RTX 2080 utilizes ~225 W under typical load conditions^66^, whereas the Kinova robotic arm consumes ~36 W (ref. ^67^). In our scenarios, each task runs for up to 4 min. Utilizing the EPA’s average conversion factor of ~0.4 kg of CO_2_ per kWh for mixed energy sources^68^, the carbon emission for each task comes to ~0.007 kg (7 g) of CO_2_.

### Language processing

The LLM processes an image and the user’s query, systematically breaking down the complex task *L*_*T*_ into a sequence of steps {*L*_1_, *L*_2_,…, *L*_*N*_}, where each step *L*_*i*_ may depend on the completion of the preceding steps. The sequence of steps is critical, and dependencies exist between steps; for example, if an object (for example, a mug) is required but not found, then potentially a cupboard should be opened.

The environmental data gathered from the initial image input are key in decomposing the abstract task. For instance, when asked to make a beverage, the ingredients present in the environment are critical in deciding which drink to make, and the visual information can help identify possible locations. The interface was facilitated by GPT-4, which ran under the instruction to write and dispatch code to a robot via the server platform. The process was assisted by a knowledge base containing code examples and allowed continuous communication with the robot. The curated knowledge base contained validated examples of low- and high-order actions that incorporate known uncertainties. Including these motion examples is key to enabling the robot to handle numerous scenarios and complete long-horizon tasks. High-level motion primitives or policies can compress multiple known uncertainties into a single function, reducing the need for extensive code writing. RAG allowed the knowledge base to be comprehensive without sacrificing performance. The system interacted with the ROS and communicated via a low-latency connection provided by the EC2 server through JSON action queries and responses.

The dependency among tasks is expressed through conditional probabilities such as *P*(*L*_2*A*_, *L*_2*B*_∣*L*_1_), which specifies the likelihood of progressing to tasks *L*_2*A*_ or *L*_2*B*_ following the successful execution of task *L*_1_. This helps in planning the sequence of steps, ensuring the robot can adapt its actions based on real-time feedback. The LLM generates executable code that is sent to the server, based on the instructions (prompt) and a knowledge base containing examples. The code is run on the ROS in a secure environment that only has access to predefined functions, thereby ensuring safety in task execution.

### RAG

A key feature of our system is the deployment of RAG. RAG integrates user queries with information from a continually updated, curated knowledge base, optimizing the output of the LLM. This approach allows the model to follow code examples provided in the database, ensuring accuracy, reliability and scalability as the knowledge base evolves.

We used vector RAG, which involves using an encoder to embed the query (*q*) and segments of the knowledge base ({*s*_1_, *s*_2_,…, *s*_*m*_}), known as chunks, into vector representations. Chunks were then compared with the query based on cosine similarity, and the top *k* chunks were selected as contextually relevant information for generating responses. Alternative retrieval techniques that can be used within our framework include traditional RAG (keyword-/rule-based RAG) or hybrid retrieval methods.

The RAG pipeline can be customized by selecting different document stores (the medium in which the knowledge base is stored and organized). In our experimental test, we used the inbuilt OpenAI RAG process and organized our curated knowledge base in a markdown file as the document store. However, a range of other RAG approaches can be used in our framework, utilizing tools like Haystack^56^ and Vebra^57^. These tools allow users to select a range of document stores—from ‘markdown files’ for simple text-based knowledge to ‘Elasticsearch’ for complex, indexed data—along with specific embedders, retrievers and chunking techniques, as well as the LLM itself.

### Vision system

Grounded-Segment-Anything was used as the language-to-vision model to create a 3D voxel that highlighted the positions of all objects and allowed their poses to be extracted for robotic grasping^58,69^. This enabled (1) the generation of object-specific bounding boxes, (2) the manufacture of segmented masks via MobileSAM and (3) the creation of voxels that encapsulate the detected objects. The voxels allowed target object poses to be extracted.

### Force module

To ensure accurate measurements in force-rich applications, we calibrated the ATI force sensor to compensate for gravitational forces, ensuring it registered zero in the absence of external forces. This calibration is key for accurately predicting the external forces applied to the end-effector. The process involved sequentially zeroing the force sensor on one axis, rotating the sensor and then zeroing on the next axis. The local forces were transformed into the global plane to estimate the upward force at different rotations *F*_global_ = *T*_end_effector_to_robot_base_ × *F*_local_, where *F*_global_ is the force vector in the global (robot base) coordinate frame, *T*_end_effector_to_robot_base_ is the transformation matrix from the end-effector’s frame to the robot’s base frame and *F*_local_ is the force vector in the local coordinate frame of the end-effector. We explored various methods, such as moving the sensor’s position and orientation and using polynomial functions for calibration. However, the simpler calibration method was found to be the most effective.

To estimate the flow rates, we assumed a condition of static equilibrium and maintain slow operational speeds during pouring. Mathematically, this is represented as *F*_up_ ≈ *mg* and Δ*F*_up_ ≈ Δ*mg*. In situations involving variable acceleration, the relationship between forces and flow rates becomes more complex. It necessitates a dynamic model that accounts for varying inputs, such as flow rates, container’s centre of mass and inertia of the end-effector, to map dynamic force inputs to the pouring flow rates.

The system continuously manages force vectors along three axes, adjusting the applied force based on the criteria within its knowledge base. The LLM dynamically selects the necessary force magnitudes and directions tailored to meet specific downstream task requirements. For example, the knowledge base may specify varying force magnitudes to be applied depending on the object characteristics or task demands. This approach enables the system to adjust its actions autonomously to align with a broad range of operational criteria.

### ROS operation

In this work, we initiated the robotic processes by launching a Kinova ROS Kortex driver. This established a node that enables communication within the ROS network and the Kinova Gen3 robot. The node publishes several topics that subscribers can access, and it provides services that can be called to modify the robot’s configuration. The base joints are updated at a frequency of 40 Hz. Concurrently, the Robotiq 2F-140 mm gripper node is activated at 50 Hz. The node sets up a communication link with the gripper via a USB connection, and it initiates an action server that enables precise control of the gripper and facilitates the exchange of operating data.

A vital element of our robotic system is the vision module node. A ‘classes’ variable is used to identify the target pose of selected objects within the environment. This variable can be dynamically updated, thereby allowing the system to adapt to changes in the scene. The pose coordinates of the objects, as established by the ‘classes’ variable, are published approximately at every $\sim \frac{1}{3}$ Hz. This is largely due to the processing time of Grounding DINO in detecting objects and establishing the bounding boxes. Moreover, we used an AprilTag to determine the position of the camera relative to the robot’s base. This is represented as *P*^R^ = *T*_AR_ × (*T*_CA_ × *P*^C^), where *P*^C^ is the point in the camera frame, *T*_CA_ is the transformation matrix from the camera frame to the AprilTag, *T*_AR_ is the transformation matrix from the AprilTag to the robot’s base and PR is the point in the robot’s base frame.

In parallel, a force node is launched at a frequency of 100 Hz and provides multiaxis force and torque readings, localized to the ATI force transducer. The readings are transformed using a quaternion-based 3 × 3 rotation matrix to align with the global base frame of the robot, providing raw and averaged values over the last five time steps across fixed degrees of freedom. It calculates forces in the global frame of the robot base using the rotational matrix, calculated from the kinematic data.

ROS facilitates the continuous processing of multimodal feedback data from the language processing, vision systems, force metrics and joint end-effector positions. The motions operate on a foundational six-degrees-of-freedom twist command, which controls velocity and the variable speed and force gripper procedures for opening and closing. This enables the integration of hard-coded safety constraints, such as maximum velocity and force limits, as well as workspace boundaries.

The linear velocities were clamped within ±0.05 m s^–1^ and the angular velocities were clamped within ±60° s^–1^. End-effector forces were also limited to 20 N. This is coded into the fundamental motion primitives; therefore, error in the language model will not override this. The end-effector is also clamped within the predefined workspace bounds of *x* = [0.0, 1.1], *y* = [–0.3, 0.3] and *z* = [0, 1.0]. This is checked in future time steps by a publisher at a frequency of 10 Hz.

## Supplementary information

Supplementary Information Supplementary Sections 1–3 and Table 1.

## Data Availability

The dataset used in this work is available in an open-source GitHub repository at https://github.com/ruaridhmon/ELLMER.

## Code availability

The code supporting this study is available via GitHub at https://github.com/ruaridhmon/ELLMER and has been archived in Zenodo at 10.5281/zenodo.14483539 (ref. ^70^).
