# Supplementary material for: Embodied large language models enable robots to complete complex tasks in unpredictable environments
Source: Nat Mach Intell. 2025 Mar 19;7(4):592–601. doi: 10.1038/s42256-025-01005-x (PMC12088599; doi:10.1038/s42256-025-01005-x)
Supplement: Supplementary file 1 — Supplementary Sections 1–3 and Table 1. [file 42256_2025_1005_MOESM1_ESM.pdf]

nature machine intelligence

Supplementary information

https://doi.org/10.1038/s42256-025-01005-x

Embodied large language models enable robots to complete complex tasks in unpredictable environments

In the format provided by the authors and unedited

Supplementary Information

1 State-of-the-art approaches, limitations, and advantages of ELLMER

Making a cup of coffee in an unconstrained kitchen is a challenge to existing robot systems because domestic environments lack the controlled object placement typical in industrial automation settings. The layout of homes is constantly changing, inhabited by individuals who frequently alter their surroundings. These ever-evolving conditions pose significant challenges for robotic systems, which need to continuously adapt for successful interactions with objects in such settings (7–9). Robots in these environments are required to manage tasks over extended periods (long-time horizons), and integrate force and visual feedback as a strategy to mitigate uncertainties, including environmental changes caused by human activity or sensor noise (8, 9). Significant advances in robot manipulation - a fundamental research problem - have been made over the past decades. However, traditional approaches often have limited ability to adapt to novel environments because of their reliance on predefined motion primitives (13). Motion primitives remain useful but major progress has been made through the use of powerful alternative techniques including reinforcement learning and imitation learning (19–23, 53, 54).

RL is a powerful technique and has an important role to play in the development of intelligent machines. However, RL requires extensive data collection and training which can be costly (24) unless simulators are used (55). Moreover, the simulators used for RL system training frequently encounter instabilities and often struggle to efficiently and accurately replicate the subtleties of real-world physics in dynamic scenarios (56–58), such as the manipulation of fluids or granular materials (16, 34, 35). While RL has proven effective for simpler tasks, such as box pushing (59), its application over a wide range of complex and varied scenarios is less successful. Techniques to address these issues include domain randomisation, meta-learning,

and extensive training over a wide range of environments (60, 61). However, these methods still struggle to adapt to a wide variety of tasks beyond their original training environments, highlighting a persistent challenge in creating broadly applicable and adaptable RL systems. Imitation Learning (IL) is an alternative method through which machines can be trained to interact skillfully with their environment. IL is adept at capturing subtle behaviours and can produce 'human-like' motions, but it struggles with distribution shift, requiring extensive diverse data to be truly effective beyond the specific data collection conditions (62). Moreover, many IL models require considerable additional training data to account for various unexpected changes in the environment, limiting robots to primarily replicate the learnt tasks without dynamically adapting to uncertainties.

The current constraints on RL and IL techniques strongly suggests that there is a need for other methodologies to be used in the development of intelligent robots. Large Language Models (LLMs) have been identified as a particularly useful approach in this regard. A substantial body of work has explored the application of pre-trained language models for embodied agents (15, 24, 25, 27, 42, 43, 63–68). This work has achieved impressive results in enabling robotic manipulation systems to comprehend context and apply their robotic skills across a wide range of tasks, predominantly those with short time-horizons (e.g., pick-and-place operations) across various scenarios. Thus, the emergence of Large Language Models (LLMs) offers opportunities to overcome traditional limitations in robotic capabilities (24, 29, 31, 69, 70). LLMs introduce versatility and provide decision-making capabilities that were previously unattainable, enabling robots to manipulate objects in complex environments with enhanced adaptability (24).

Current LLM-driven approaches often rely on detailed prompts to correctly guide robot actions. However, the reliance on these lengthy prompts and inefficient feedback mechanisms

means systems often struggle with complex, long-horizon tasks that require a diverse set of skills across various scenarios (24,32). One powerful approach made possible through LLMs is providing robots with the ability to access and utilise extensive ‘knowledge bases’. Leveraging knowledge bases to enhance LLMs’ potential for robotic applications has already shown substantial benefits (67). This method enables robots to access and retrieve relevant action examples and information, assisting precise response generation. Nonetheless, these approaches have been limited to date by the size and thus diversity of the knowledge base because of decreases in performance when large knowledge bases are added directly into LMM’s context windows (15, 67). Retrieval-augmented generation (RAG) represents a significant advancement in overcoming this limitation that could allow robots to take full advantage of LLMs (32).

We developed the Embodied Large Language Model Enabled Robot (ELLMER) framework to allow diverse developments across artificial cognition and robotic control to be combined and ultimately bring together a wide variety of techniques (including RL and IL). Current state-of-the-art approaches, such as ‘code as policies’, focus on processing perception outputs but have not incorporated force feedback or a curated knowledge base – creating a barrier to the evolution of intelligent machines. In contrast, ELLMER is an important first step towards enabling adaptive robotic behaviour that can utilise higher order cognitive reasoning in complex environments in real-time. The addition of force feedback with vision in control loops provides a step change in the ability of robots to perform delicate manipulation tasks. These sensorimotor abilities are further enhanced through the knowledge of the world obtained from the LLM. The addition of RAG within an ‘intelligent machine’ framework allows domain-specific information retrieval from a knowledge base during task execution, improving decision-making and adaptability. RAG is established in natural language processing, but it has not been previously applied to dynamic robot task adaption and thus ELLMER represents a key innovation capable

of accelerating the development of intelligent machines. The curated knowledge base enhances the LLM's performance by tailoring information retrieval to the specific needs of the task being undertaken by the robot and this ensures high quality, contextually relevant outputs. In this context, RAG can be seen as providing a cultural milieu of knowledge from which a robot can draw. Notably, this mirrors the 'intelligence' afforded to humans through the cultural transmission of knowledge.

2 Accuracy when drawing shapes

We found that utilising force in the z-axis was important for enabling the pen to apply the desired pressure, even amidst uncertainties in the object's surface position relative to the camera. We found that the quality of DALL-E-generated images varied with the specificity of the input prompt and the capabilities of the language-to-vision model. A fixed low-velocity of $0.01m/s$ was found to be effective when drawing the designs. Figure 5 illustrates the robot's accuracy at this constant speed. Table 1 provides details on the time it took the robot to complete each shape and its similarity to the original (measured using the Jaccard Index). Maintaining a low speed helped ensure precise tracing by preventing overshoots as the robot navigated through the waypoints.

Table 1: Accuracy of the plotting methods.

**Shapes**	**Time to completion (S)**	**Completeness** (%)
Random Animal	100.09	98.56
Random Food	79.34	99.45
Random Plant	115.65	98.03

3 ELLMER and its future development

Our study was conducted using a Kinova Gen3 7-degree-of-freedom robotic arm. Nevertheless, the underlying principles and architecture of the framework are designed to be largely hardware-agnostic, allowing for potential compatibility with a range of robotic platforms beyond the Kinova arm. Scalability is a critical asset of our framework, encouraging a collaborative approach to advancing intelligence machines (as opposed to the current situation of isolated fragmented projects throughout the field of robotics). This collective methodology has the potential to encourage progress by promoting the sharing of modules and knowledge across different projects.

Several general hardware components are essential to implement the framework on other robotic platforms. These components include a robotic manipulator with sufficient degrees of freedom to perform manipulation tasks, force/torque sensing capabilities (either embedded in the robot's joints or mounted externally for tasks requiring fine force control), a vision system compatible with standard sensors (such as RGB-D cameras to provide visual feedback for object detection and environment mapping) and an appropriate end-effector capable of interacting with various objects. The hardware used will shape the curated knowledge base and accessible functions. Some of the functions are robot-agnostic, allowing for flexibility across robotic systems (e.g., modules that control the velocity of the end-effector are independent of the specific robot hardware). Other modules are tailored to the robot and its environment, requiring specific configurations. This ensures that ELLMER can generalise across systems, but it can also use the specific capabilities of an individual robot. This approach fosters collaboration and shared expertise through a central framework - aiding the continuous development and improvement of intelligent machines and combining expertise across the field.

Intelligent machines will need to reply on sensorimotor systems, in the same way that human

intelligence is grounded in our sensorimotor capacity of utilising force and vision to navigate issues of accuracy and durability. These 'sensorimotor' systems enable machines to interact adaptively and precisely with their environment. However, repeated use may lead to sensor degradation, drift, or wear, affecting performance over time. To ensure consistent accuracy and durability of the sensors, regular calibration, maintenance, and use of industrial-grade sensors will be essential. Nevertheless, the existence of 'intelligence' and feedback from force and vision within the ELLMER framework means that systems will be better equipped to detect inaccuracies and wear-and-tear and thus act to ensure these issues do not go undetected and cause unwanted consequences 'in the wild'.

It is important to note that the system has not been tested with fragile or deformable materials. Thus, the rigid gripper we used might damage objects made from delicate or soft materials. To handle such materials, more adaptable grippers – such as soft robotic grippers, underactuated hands, or grippers with compliant materials – are needed to provide the necessary flexibility and compliance. Incorporating tactile sensors and employing soft robotic techniques would enhance the robot's ability to grip with enough force to secure an object but without causing damage. This is an important field of future research, and our hope is that ELLMER will allow advances in such research to be incorporated efficiently into the future design of intelligent robots. For example, ELLMER allows robots to leverage LLMs to provide an accurate "model of the world", allowing the robot to infer the physical properties of objects – and by using force feedback in combination with these models, the robot can ensure the forces applied are suitable for the object's properties. Implementing these outcomes would require additional modules or specific code that can model and control these complex scenarios, potentially relying on advanced control algorithms or reinforcement learning methods to manage the interactions with deformable materials. ELLMER provides an elegant way of incorporating such modules and generating the

appropriate code.

We recognise that although our experiments were conducted in environments involving the manipulation of liquids and solid objects, the use of a rigid mechanical design significantly influences the robot's effectiveness across diverse settings. In our study, we employed an articulated Kinova arm equipped with a gripper and force sensor, composed of materials such as aluminium alloys, advanced plastics, rubber, silicones, and stainless steel, optimised for tasks requiring high accuracy and stability. The mechanical properties of this arm clearly influence the robot's performance. Therefore, research into soft robotics is important if intelligent machines are to realise their potential. Once again, we note that the integration of force and vision feedback improves the robot's ability to address these challenges. The robot can use real-time force feedback to adjust its grip and applied force, optimising its interactions based on both its own mechanics and the specific object properties. This feedback minimises the risk of inefficiency or damage, particularly when handling fragile or different materials.

For reliable task execution, the robot must select the appropriate modules from the database, taking into account its own payload and operational limitations. The modules can be designed to apply appropriate forces, considering the robot's material constraints, payload capacity, mechanical resilience, and the mechanical properties of the objects it interacts with. This is important for reliability, especially for performance in tasks with manipulation of liquids and solids under controlled conditions. In the longer term, there is a need to expand robots' capabilities to handle a broader array of tasks in varied domestic and industrial settings to enhance the robot's operational versatility. The development of a modular system architecture with a comprehensive library of task-specific modules – each with defined inputs and outputs - would enable seamless integration of new functionalities. This approach could be further improved by creating a

domain-specific language for task configuration, promoting collaboration between researchers and companies through a centralised database of models.

In parallel to human intelligence – where sharing of knowledge ('culture') is an essential component of the human evolutionary success story – ELLMER promotes a collaborative effort across researchers. Researchers can combine their work and drive innovation by connecting and coordinating efforts through a centralised database. ELLMER enables the robot to intelligently select and apply task-specific modules, enhancing its adaptability and reliability across various applications. The framework can continuously improve by leveraging shared expertise, refining robot capabilities to meet emerging challenges.

Our demonstrations, such as making coffee and decorating a plate –involve complex sequences and multiple modalities – highlighting the sophistication of the system. However, these tasks represent just a subset of possible applications. The framework's modular and flexible design allows scalability to more complex applications by expanding the knowledge base with additional motion primitives and policies. Future work will involve testing in dynamic environments, integrating advanced control algorithms, and enabling the robot to autonomously acquire new skills, demonstrating the framework's capacity for tackling diverse and challenging tasks.

We recognise that LLMs and RAG will continue to evolve, and ELLMER is built to take advantage of the developments in these technologies. A challenge is ensuring that the knowledge base is continuously updated, particularly as new robot designs and capabilities emerge. To address this, ELLMER can incorporate continuous updates and maintenance of the knowledge base – for example if the robot design changes. ELLMER allows future work to integrate

adaptive learning capabilities, enabling the system to dynamically update on the basis of new experience.

In our study, we noted that the vision system sometimes struggled with occlusions and distinguishing visually similar objects, particularly in cluttered environments. This can lead to inaccuracies in object detection and pose estimation. However, just as humans demonstrate intelligence by relying on multiple senses, ELLMER integrates vision and force feedback to compensate for noise or signal loss from another source. ELLMER allows the incorporation of future enhancements (such as more advanced perception algorithms that can better handle occlusion and ambiguous visuals). ELLMER also supports the combination of visual data with other sensory inputs (e.g., tactile feedback) and this can improve object recognition and manipulation, leading to more robust performance in complex scenarios.

In conclusion, while ELLMER establishes a framework in leveraging rigid robotic systems for precise manipulation tasks, we anticipate that future research will drive great improvements in durability, operational scenario opportunities, scalability and dynamic environment synchronisation. This will be key for enhancing the adaptability and utility of integrated AI and sensorimotor systems in real-world applications.
